# Risk of Gluten Cross-Contamination Due to Food Handling Practices: A Mini-Review

**DOI:** 10.3390/nu16081198

**Published:** 2024-04-18

**Authors:** Renatta Pereira B. Damasceno, Renata Puppin Zandonadi, Marcela Mendes, Luis Carlos Cunha Junior, António Raposo, Edite Teixeira-Lemos, Cláudia Chaves, Priscila Farage

**Affiliations:** 1 School of Nutrition, Federal University of Goiás (FANUT/UFG), Goiânia 74690-900, Goiás, Brazil; renattad.nutri@gmail.com (R.P.B.D.); mendesmarcelam@gmail.com (M.M.); 2 Department of Nutrition, College of Health Sciences, University of Brasília (UNB), Brasília 70910-900, Federal District, Brazil; renatapz@unb.br; 3 School of Agronomy, Federal University of Goiás (EA/UFG), Goiânia 74690-900, Goiás, Brazil; cunhajunior.l.c@ufg.br; 4 CBIOS (Research Center for Biosciences and Health Technologies), Universidade Lusófona de Humanidades e Tecnologias, Campo Grande 376, 1749-024 Lisboa, Portugal; 5 CERNAS Research Centre, Polytechnic University of Viseu, 3504-510 Viseu, Portugal; etlemos3@gmail.com; 6 ESSV, Centre for Studies in Education and Innovation (CI&DEI), Polytechnic University of Viseu, 3504-510 Viseu, Portugal; claudiachaves21@gmail.com

**Keywords:** celiac disease, gluten, gluten-free, contamination, gluten cross-contact, gluten-free diet

## Abstract

Celiac disease (CD) is an autoimmune disease triggered by the ingestion of gluten in genetically predisposed individuals, affecting 1.4% of the world population. CD induces an inflammatory reaction that compromises small intestine villi, leading to nutrient malabsorption, and gastro and extraintestinal manifestations. Although other treatment approaches are being studied, adherence to a gluten-free diet (GFD) is the only effective intervention to date. Despite this, about 50% of patients experience persistent inflammation, often associated with unintentional gluten ingestion through contaminated food. There are regulations for labeling gluten-free foods which specify a limit of 20 mg/kg (20 ppm). The risks of gluten cross-contamination above that level are present throughout the whole food production chain, emphasizing the need for caution. This review explores studies that tested different procedures regarding the shared production of gluten-containing and gluten-free food, including the use of shared equipment and utensils. A literature review covering PubMed, Scielo, Web of Science, VHL and Scopus identified five relevant studies. The results indicate that shared environments and equipment may not significantly increase gluten cross-contamination if appropriate protocols are followed. Simultaneous cooking of gluten-containing and gluten-free pizzas in shared ovens has demonstrated a low risk of contamination. In general, shared kitchen utensils and equipment (spoon, ladle, colander, knife, fryer, toaster) in controlled experiments did not lead to significant contamination of samples. On the other hand, cooking gluten-free and gluten-containing pasta in shared water resulted in gluten levels above the established limit of 20 ppm. However, rinsing the pasta under running water for a few seconds was enough to reduce the gluten content of the samples to less than 20 ppm.

## 1. Introduction

Celiac disease (CD) is a permanent autoimmune disorder triggered by the ingestion of gluten in genetically predisposed individuals [1,2]. Gluten is present in cereals such as wheat, barley, rye, and oats. However, the consumption of oats seems to be safe for most celiacs but may be immunogenic in a small percentage of them, which are sensitive to some oat cultivars. The variability in the tolerance of oats may be related to differences in cultivars, harvesting, and amount of oat ingestion. There is no consensus in the literature about safety and intervals for monitoring tests and symptoms after introducing gluten-free oats into a gluten-free diet [3]. Some countries do not recommend oats consumption due to the risk of gluten contamination and unintentional ingestion [3]. In particular, it has been reported that oat consumption should be considered safe in many countries, though it may be discouraged in developing countries where contamination could be widespread, as recently well-described in a comprehensive comparison of current international guidelines [3].

Celiac disease is underestimated because of poor awareness among the population and physicians; narrow access to diagnosis; inadequate use (or interpretation) of serological tests; absence of standardized diagnostic protocols; and inadequate expertise in histopathological interpretation [3]. Despite that, CD diagnosis has been increasing in recent years due to increased awareness, testing, and the rise in autoimmunity (shown by seroprevalence in apparently asymptomatic individuals) [4]. The worldwide prevalence of CD is 1.4% [5]. As a determining factor for CD, the ingestion of foods containing gluten (wheat, rye, barley and all its derivatives) causes an inflammatory reaction that compromises the villi of the small intestine, impacting the absorption of nutrients [6,7]. The diagnosis of CD is determined by clinical suspicion, signs and symptoms, serological tests, intestinal biopsy and positive response to a gluten-free diet [8].

Besides CD, other diseases are associated with gluten ingestion, such as wheat allergy and non-celiac gluten sensitivity (NCGS) [9]. Considering these three conditions, it is estimated that around 10% of the population must adopt a gluten-free diet (GFD), reinforcing the importance of studies like this [10,11]. Although it may seem simple, following a GFD is complex, given the widespread presence of wheat and other cereals containing gluten in foods and preparations consumed worldwide [11]. Due to the desirable sensory characteristics that gluten provides to food, such as elasticity, firmness, cohesion, moisture and uniformity, cereals with gluten can be incorporated into most meals daily [12,13]. Celiac disease presents a broad spectrum of clinical manifestations, resembling a multisystemic disorder instead of an isolated intestinal disease [4]. In this sense, the CD does not have a defined pattern and patients may present one or more gastrointestinal or extraintestinal symptoms or even be asymptomatic [14]. Generally, the most common clinical symptoms of CD include diarrhea and/or constipation, abdominal distension, and malnutrition, among others [15,16]. Atypical or extraintestinal manifestations can appear in the form of anemia, osteoporosis, dermatitis herpetiformis, neurological disorders and so on [17,18]. There may also be possible complications, including poor infant growth, infertility, and malignant diseases [6,19,20].

Currently, intervention in CD is mainly nutritional and dietary, with the only treatment option being strict adherence to a GFD to contain inflammation and the progression of complications, as well as improvement of serological parameters and clinical symptoms [21,22]. However, 50% of celiac patients show persistent signs of inflammation, such as villous atrophy, which may be associated with the unintentional ingestion of gluten in contaminated foods [23,24]. Therefore, cross-contamination in naturally gluten-free foods is a concern for these subjects [25]. When adopting a GFD, celiac individuals must carefully check the labels of all food products and dietary ingredients used for culinary preparations to guarantee a complete gluten restriction [26,27]. Therefore, the burden of CD is related not only to the clinical manifestations and symptoms but also to the cost of diagnosis investigation and monitoring; social burden; the cost of gluten-free foods compared to their counterparts and the fear of unintentional gluten intake.

National and international regulations specify limits, definitions and even standards for gluten-free food labeling [28]. Foods such as meat, fish, dairy products, vegetables, nuts, fruits, rice, amaranth, and corn do not naturally contain gluten, but may present gluten traces resulting from cross-contamination [29]. According to the WHO/FAO Codex Alimentarius Commission, “gluten-free foods are those in which the gluten level does not exceed 20 mg/kg (ppm) in total” [26,28,30]. Cross-contamination might occur in any step of the food production chain, from planting to the final preparation, whether at home, restaurants and even within the food industry, which may put celiac individuals at risk [31].

Given the considerable burden of cautiousness and awareness concerning the presence of gluten placed on patients and their families, as well as on restaurants and food industries responsible for food production, a better understanding of the risks associated with the practices that may lead to gluten cross-contamination is necessary [32]. In a pilot study carried out in 2016, Miller et al. tested the following hypotheses regarding the safety of producing gluten-free food in commercial kitchens that use wheat flour: (i) increasing the duration of exposure to wheat flour would increase gluten contamination; (ii) increasing the distance between the area of gluten-free food preparation and wheat flour handling would reduce gluten contamination; (iii) the use of a fume hood would reduce gluten contamination; and (iv) the use of a barrier segregating the production area of a gluten-free meal and the area of wheat flour use would reduce gluten contamination. After the analyses, they reported that naturally gluten-free foods may be safely prepared simultaneously with wheat-based flours in shared commercial kitchens, as long as appropriate protocols for hygienic control are followed [33].

Currently, there is growing worry about the ingestion of gluten-contaminated foods by individuals with CD. Studies around the world have identified gluten contamination both in industrial food products and food service meals [34]. Despite being a valid and important concern, hypervigilance and excessive fear regarding the safety of GFD are associated with food anxiety, fatigue and feelings of social exclusion, which directly impact the quality of life of these individuals [35]. Therefore, the risk of gluten contamination must be better elucidated, particularly in relation to the degree of contamination obtained in the final product as a result of certain culinary techniques and cooking methods, including the sharing of utensils and equipment.

The aim of this review was to identify studies in the literature that evaluated different procedures regarding shared production of gluten-containing and gluten-free food and to discuss the risk of contamination arising from these practices.

## 2. Materials and Methods

### 2.1. Search Strategy

To prepare this mini-review, a literature search was carried out using the following databases: PubMed, Scielo, Web of Science, BVS and Scopus. The following search strategy was used: ((Gluten) OR (Celiac Disease) OR (Coeliac Disease) OR (Diet, Gluten-Free)) AND ((contamination) OR (food contamination) OR (Equipment Contamination) OR (Detection) OR (test)). The final database search was carried out on 18 December 2023.

### 2.2. Eligibility

We included experimental studies that tested the use of shared environments, equipment, and utensils for the production of gluten-free and gluten-containing food to assess possible cross-contamination. As inclusion criteria, the following was defined: experimental studies that analyzed gluten content in gluten-free food through the Codex and the Association of Official Analytical Chemists (AOAC) recommended methods for gluten analysis. The exclusion criteria were: (i) reviews, letters, books, conference abstracts, editorials, and opinion articles; (ii) studies that performed unrecognized gluten analysis methods; and (iii) studies that did not assess gluten content in the final product. There were no publication restrictions regarding date, language, or country. Experimental studies were retrieved in the literature search, and those that met the eligibility criteria were included to define and discuss the risk of gluten cross-contamination.

### 2.3. Studies Selection

Initially, titles and abstracts were screened to identify duplication and eligibility. Studies that did not meet eligibility criteria were excluded. The selected articles were read in full in the following phase to confirm eligibility. Furthermore, to identify additional potentially eligible studies, the reference lists of included studies were manually searched. The Mendeley software (version 1.19.5) was used for duplicate detection, title/abstract screening, and management of references selected for full-text reading.

### 2.4. Data Collection

The available data published in each study (author, year of publication, title, keywords, country, type of study, objective of the study, methods, utensils tested, repetitions, test used to analyze gluten content, methods of cleaning, results, limitations according to authors and other limitations) were collected using a standardized data extraction form.

## 3. Results and Discussion

**Figure 1:**
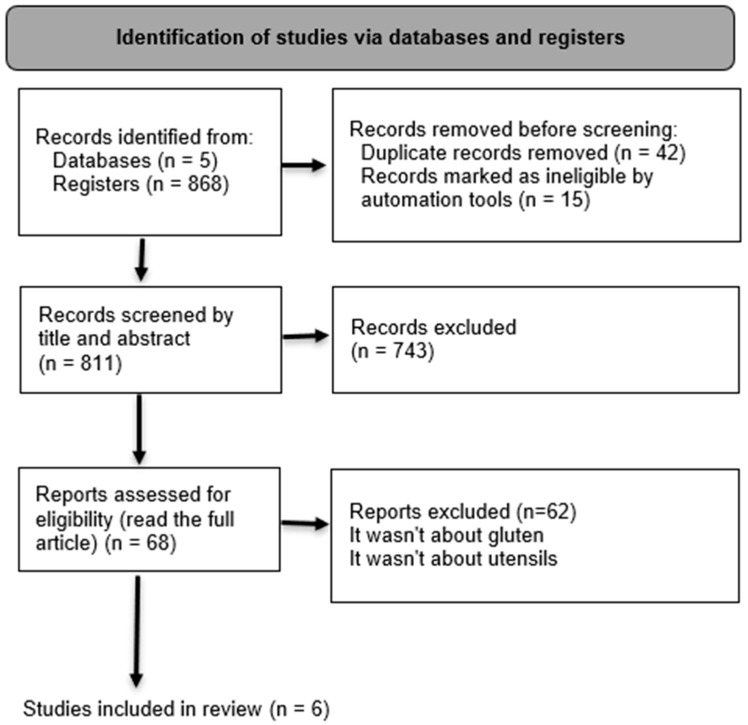
Flow diagram for study identification and selection (PRISMA, 2021).

A total of six studies that evaluated food preparation methods and the degree of gluten contamination in the final product/meal were selected (Figure 1). In this review, the recommended cutoff point of 20 ppm for classifying gluten contamination was adopted [26].

Table 1 presents the main characteristics and results of the articles discussed in this review. The studies were published between 2016 and 2021, and the majority were conducted in the USA [36,37,38,39], one was performed in Switzerland [40] and another in Italy [41]. All studies adopted the ELISA (Enzyme-Linked Immuno Sorbent Assay) method to quantify gluten, which is the gold standard technique for gluten analysis. The Ridascreen Gliadin R5 Sandwich ELISA kit from R-Biopharm was the most used laboratory kit [42]. One study also used the PCR (Polymerase Chain Reaction) method in the analysis.

Most studies evaluated cross-contamination through sharing utensils and equipment for handling food with and without gluten (n = 4). One study assessed gluten cross-contamination through shared cooking water for pasta. The food samples analyzed in these studies were: pasta, pizza, bread, cupcakes, French fries, tater tots, tortilla chips, and popular spreads for sandwiches (jelly, mayonnaise, and peanut butter). As for utensils, studies have tested the use of a knife, colander, spoon, ladle, and pans as possible sources of cross-contamination. Regarding equipment, studies included experiments with a toaster, oven, and electric fryer (Table 1).

Vincentini et al. [41] conducted a two-stage study with the aim of identifying a safe procedure for cooking gluten-free pizza. In the first stage, three shared pizza production scenarios were tested in a bakery school kitchen: in procedure one (i), pizzas with and without gluten were baked simultaneously in the same oven; in the second procedure (ii), pizzas were baked alternately in the same oven, with a batch of gluten-free pizzas followed by a batch of wheat-based pizzas; and in the third procedure (iii), wheat-based and gluten-free pizzas were cooked in different ovens, one dedicated to gluten-free pizzas and another dedicated to gluten-containing pizzas. In the second stage, gluten-free pizzas prepared in five different pizza houses that serve both gluten-containing and gluten-free pizzas were collected for posterior analysis. The gluten-free pizza samples collected were produced using the same procedures mentioned above (procedures i, ii and iii). Moreover, researchers also tested, on a specific day, the use of gluten-free flour to roll out both gluten-free and wheat-based pizzas in the pizza houses, as opposed to the traditional use of wheat flour for rolling out regular wheat pizza. This procedure was applied to assess if using only gluten-free flour in the shared production area would lead to a lower risk of cross-contact, since aerosolized flour in the kitchen may be a source of gluten-contamination.

Among the total of 154 analyzed pizzas, the authors found only one contaminated sample of pizza cooked in the bakery school kitchen following procedure one (i). All the other pizzas produced, both in the training school kitchen and in the five participating pizza houses, showed a gluten concentration below 20 ppm. These results suggest that sharing the oven simultaneously or alternately does not present a significant risk of gluten cross-contamination. It is important to clear out that the staff from all pizza houses had been trained on safe gluten-free production. These findings are promising in the sense that, apparently, producing gluten-containing and gluten-free foods in the same environment, and using the same equipment, will not necessarily trigger cross-contamination, as long as employees are properly trained. The authors mention that it was not possible to draw definitive conclusions regarding the impact of using wheat flour for rolling out wheat pizzas on gluten cross-contact in gluten-free pizzas due to the methodology of the study. Nevertheless, they strongly suggest using gluten-free flour to roll out both types of pizza, as wheat flour residue can be aerosolized when handled, which can lead to contamination of gluten-free pizzas and other gluten-free raw materials in the various stages of the production process [41].

In the study by Studerus et al. [40], the degree of gluten cross-contamination due to the sharing of household kitchen utensils was assessed. Ten standardized scenarios were tested and replicated three times, to quantify the gluten content in the samples (pasta and bread) resulting from sharing knife, colander, and ladle. The kitchen was divided into ‘gluten-containing’ and ‘gluten-free’ areas, with a space of 4 m between them. The following procedures were performed: (i) using a knife to cut a slice of gluten-containing bread, in the gluten-containing area, and immediately taking it to the gluten-free area, without it being sanitized, and using it to cut the gluten-free bread; (ii) using a colander to drain the gluten-containing pasta and then using it to drain the gluten-free pasta; (iii) using a ladle to serve the gluten-free pasta in the gluten-containing area and then using it to serve the gluten-free pasta in the gluten-containing area; and (iv) placing a wooden spoon in the pan during the cooking of gluten-containing pasta and then placing it in the pan used for cooking gluten-free pasta. Surprisingly, as a result of these procedures, the gluten concentration in all samples was lower than 20 ppm.

In this study, three cleaning techniques were also tested to verify gluten and wheat DNA concentrations in clean utensils after preparing gluten-containing foods. The following procedures were followed: cleaning the colander with cold and warm water for 10 s; cleaning the colander with a clean cloth and towel; cleaning the colander with a cloth and towel containing flour; cleaning the knife with a clean cloth and towel; cleaning the knife with a cloth and towel with flour residue. All cleaning methods tested did not result in relevant contamination, presenting gluten concentrations <10 ppm [40].

In the study by Parsons et al. [37], three common cooking practices were investigated as possible triggers of gluten cross-contamination: (i) sharing the same fryer and the same oil to fry gluten-containing foods (chicken nuggets) and gluten-free foods (French fries); (ii) the use of the same toaster to prepare bread with and without gluten; and (iii) sharing a knife to apply mayonnaise, jelly and peanut butter to gluten-containing and gluten-free bread. Samples of the naturally gluten-free foods were tested by ELISA after each procedure (shared use of equipment and utensils) for gluten quantification, in duplicate. Regarding experiment (i) (frying), besides analysis carried out in the laboratory, samples of fried foods produced in fast-food restaurants were also collected and analyzed.

The authors found that the majority of the samples (93.6%) did not show significant levels of cross-contamination. There was no contamination above 20 ppm in the samples of French fries prepared in the experimental fryer, the highest value found was 12.78 ppm and the lowest was 4.56 ppm. Among the 74 fried samples from fast-food restaurants, a single tater-tot sample showed detectable gluten levels above the established limit (21.55 ppm). Gluten-free bread samples toasted in a toaster previously used for gluten-containing bread did not show gluten levels above 20 ppm. On the other hand, gluten contamination above 20 ppm was detected in the mayonnaise and peanut butter samples. Among the 60 mayonnaise samples tested, 30 (50%) contained detectable amounts of gluten, of which 11 (18%) tested above the 20 ppm limit. Of the 60 peanut butter samples tested, 12 (20%) contained detectable amounts of gluten, of which 6 (10%) displayed gluten content above 20 ppm [37].

The study by Weisbrod et al. [36] addressed gluten transfer and the efficacy of washing methods during food preparation in three different scenarios: gluten-free pasta cooked in the same pot as gluten-containing pasta; gluten-free bread toasted in toasters also used for gluten-containing bread; and gluten-free cupcakes cut with a knife previously used to cut gluten-containing cupcakes.

In the pasta cooking scenario, gluten was detected in all samples cooked in water used for food containing gluten. Gluten levels ranged from 33.9 ppm to 115.7 ppm. However, rinsing pasta under running water effectively decreased the gluten content to less than 20 ppm. In the scenario of toasting bread in a shared toaster, results did not show gluten contamination above 20 ppm. The three samples that contained detectable gluten had very low levels, ranging from 5.1 ppm to 8.3 ppm. Regarding the cupcakes, only two samples out of 28 (7%) tested above 20 ppm. This suggests that the risk of gluten contamination when preparing gluten-free cupcakes along with other gluten-containing foods is relatively low. In regards to the washing methods, washing the pans exclusively with water, after preparing pasta containing gluten, has been shown to be as effective as scrubbing it with soap and water in preventing detectable transfer of gluten. As for using a knife to slice cupcakes, all three cleaning methods (washing with soap and water, just rinsing under running water or cleaning with antibacterial wipes) demonstrated satisfactory results in terms of the risk of cross-contact [36].

In the study by Korth et al. [38], the occurrence of cross-contamination by gluten was tested through the simultaneous cooking of traditional pasta and gluten-free pasta, in four-compartment pans in portions of 52 g (standard portion) and 140 g (portion served in restaurants). Five consecutive batches of traditional and gluten-free pasta (52 g and 140 g) were prepared in the same water. Then, both the cooking water and the gluten-free pasta were tested for gluten content. The results revealed that gluten concentrations in water samples and gluten-free pasta for a 52 g portion were adequate, remaining below 20 ppm for the five cooking batches.

However, when the authors analyzed 140 g samples of gluten-free pasta, there was a consistent increase in gluten concentrations in the cooking water. After the fourth and fifth batches, gluten concentrations in the water exceeded 50 ppm and 80 ppm, respectively. Furthermore, gluten concentrations in the 140 g gluten-free dough samples also increased, reaching almost 40 ppm after the fifth batch [38].

These results suggest that when cooking a larger quantity of traditional pasta and gluten-free pasta simultaneously, there is a greater likelihood of gluten cross-contamination. This may be especially concerning for people who are extremely sensitive to gluten, as gluten intake as high as 10 mg/day may be harmful for some individuals with CD [43]. To exceed this daily tolerance limit, a total of 500 g of food containing 20 ppm of gluten would have to be consumed, which might not be such a large amount, depending on the portion size and number of times it is eaten during the day.

Thompson et al. [39] evaluated levels of gluten cross-contamination in 20 samples of French fries collected from 10 different restaurants in California and Ohio. Two samples were collected per establishment on two consecutive Saturdays. When the samples were taken, the researcher asked the staff how the fries were prepared, whether they were prepared with wheat-containing foods and whether they shared the same oil and the same fryer. All restaurant employees said that they shared the same fryer for preparing gluten-containing foods, but they also said that neither the potatoes nor the oil contained gluten.

In this study, samples were analyzed through the ELISA R5 sandwich R7001 and ELISA R5 competitive R7021 kits, both from R-biopharm, Darmstadt, Germany [42], for the quantification of gluten levels. A microwave control was also carried out in this study.

Based on the test results, 25% of the French fry orders could not be considered gluten-free. In the sandwich ELISA R5 test, gluten was found in 9 out of 20 samples, with levels ranging from 7 to >80 ppm, where five samples tested above 20 ppm. In the competitive ELISA, the presence of gluten was observed in 3 of the 20 potato French fry samples, with levels ranging from 14 to >270 ppm, where 2 samples showed levels above 20 ppm. In the microwave control experiment, which involved mixing gluten at 60 ppm with wheat flour and canola oil, the results revealed that the unheated mixture showed an average of 64 ppm gluten when assessed by the sandwich ELISA method and 137 ppm gluten by the competitive ELISA method. On the other hand, the mixture heated to 190 °C showed an average of 55 ppm gluten using the sandwich ELISA and, respectively, <10 ppm and 16 ppm gluten using the competitive ELISA.

According to Thompson et al. [39], the results of the analysis revealed different concentrations of gluten in the French fry samples, even in samples from the same establishment, which can be explained by various factors such as the types of food previously prepared, the frequency of oil changes and the filtering system used. The authors of this study therefore suggest that further studies into the impact of these factors on the concentration of gluten in shared fryer oil merit further scientific investigation.

This review demonstrated that the majority of samples analyzed in the studies did not display gluten content above 20 ppm. The procedure that most triggered contamination above this limit was cooking gluten-free pasta in water used for gluten-containing pasta [36,38] and sharing the same fryer and oil for preparing gluten-containing and gluten-free foods [39]. Studies also suggest that, with appropriate practices and staff training, gluten cross-contamination may be minimized in shared environments. The use of common utensils and equipment appears to be safe, as long as simple cleaning measures are taken. The adoption of some precautions, such as the use of gluten-free flour to roll out the dough, adequate training, and effective cleaning, is recommended to ensure safe production of gluten-free foods in shared environments.

## 4. Conclusions

There are few studies on the risk of gluten cross-contamination due to food handling practices. However, the findings showed that some kitchen/food production procedures may display a lower risk of gluten contamination than commonly assumed, even though the care on gluten cross-contamination must be continuous. Furthermore, adopting simple measures, such as cleaning practices, avoiding direct contact between gluten-free and gluten-containing food in ovens, and not sharing the cooking water, may effectively reduce the gluten-cross contamination risk. It should also be noted that the need for dedicated kitchen utensils or designated areas for gluten-free foods may be dispensable, as long as appropriate cleaning practices are consistently applied. Further studies are necessary to confirm the procedures that put CD patients at risk of gluten cross-contamination, reducing their burden with GFD and guiding procedures to ensure the safety of food for CD individuals.

## Figures and Tables

**Table 1:** Main characteristics and results of experimental studies that analyzed gluten cross-contamination.

Author, Year	Country	Aim	Samples	Equipment/Utensils	Test
Vincentini et al., 2016 [41]	Italy	Identify safe procedures for preparing GF foods, comparing the gluten concentration of GF pizzas prepared in different shared production pizzerias, following different procedures	Pizza	Pizza oven	ELISA *
Studerus et al., 2018 [40]	Switzerland	This study was conducted to determine whether cross-contamination occurs through shared domestic kitchenware and, if so, which cleaning method is most reliable for avoiding this cross-contamination	Pasta and bread	Knife, colander, spoon, and ladle	ELISA; PCR **
Weisbrod et al., 2020 [36]	United States of America	Quantify gluten transfer when GF foods are prepared alongside gluten-containing foods. A secondary aim was to assess the efficacy of cleaning methods for kitchen equipment/utensils.	Pasta, bread, cupcake	Stainless steel pans, colander, toaster	ELISA
Parsons et al., 2020 [37]	United States of America	Determine if three common food practices lead to gluten cross-contact in gluten-free processed foods	French fries, nuggets, bread, popular sandwich spreads (mayonnaise, jelly, peanut butter)	Fryer, toaster, knife	ELISA
Korth et al., 2021 [38]	United States of America	Determine whether gluten transfer occurs between traditional and gluten-free pasta when cooked simultaneously in the same water	Pasta	Cooking pan	ELISA
Thompson et al., 2021 [39]	United States of America	To help inform consumer recommendations by assessing the gluten levels of French fries without gluten-containing ingredients cooked in fryers shared with wheat.	French fries	Fryer	ELISA

* ELISA (Enzyme-Linked Immuno Sorbent Assay); ** PCR (Polymerase Chain Reaction).
